# A brief online mindful parenting program: Feasibility and initial effects pilot in a community sample

**DOI:** 10.1007/s10826-023-02571-7

**Published:** 2023-04-01

**Authors:** Ashra Sherwood, Jessica Paynter, Lisa-Marie Emerson

**Affiliations:** 1 School of Applied Psychology, Gold Coast Campus, Griffith University, Southport, QLD Australia; 2 School of Health Sciences, University of Canterbury, Christchurch, New Zealand

**Keywords:** Mindful parenting, Parenting stress, Parenting, Online MBI

## Abstract

Mindful parenting programs are effective in reducing parenting stress. More efficient offerings may increase accessibility. The current single case study aimed to determine the feasibility, acceptability and initial effects of a brief, online mindful parenting program. Six parents, recruited from the community, completed a 4-week online mindful parenting program (Two Hearts). Feasibility and acceptability were assessed by participant program evaluation, retention, engagement with program materials (i.e., videos), and home practice. Parents completed primary outcome measurements of parenting stress, and general distress, at pre- and post-intervention, and 4-week follow-up. Individual level reliable change index and clinically significant change were calculated for outcome measures. All parents were retained through the study; all participants reported obtaining something of lasting value from the training. Program adherence varied over time. At post-intervention, four parents reported 40–50 minutes practice per week; two parents reported 10–15 minutes practice per week. At follow-up, 50% of parents reported 30–50 minutes practice per week. Three parents showed a reliable reduction in parenting stress; two of these parents demonstrated clinically significant change. Improvements in parent general distress were indicated in half the sample. Two parents experienced a clinically significant increase in parenting stress and/or general distress. In conclusion, the Two Hearts program demonstrated good acceptability, and may be a feasible and effective program for some parents. Program adherence and dosage require further investigation. The role of acute stressors (e.g., COVID-19) must be also considered.

## Highlights

- A brief (4-week) online mindful parenting program demonstrates good acceptability for parents.
- Such an intervention may reduce parenting stress and general distress in community populations.
- Issues of feasibility, adherence and dosage in online mindful parenting programs require further investigation.

Many people report, that while often rewarding, parenting is also a source of stress; this stress can negatively impact parenting skills (Bögels et al., 2010), marital functioning (Robinson & Neece, 2015), and parental mental health (Deater-Deckard, 2004). In addition, parenting stress is associated with increased child behavioral issues (Barroso et al., 2018). Provision of effective interventions and support that target parenting stress is thus important. Mindfulness—or the process of bringing intentional, non-judgmental awareness to the present moment (Kabat-Zinn, 1990) – and its application to parenting in the form of mindfulness-based interventions (MBIs), may be one possible solution. MBIs for parenting aim to improve the parent-child transactional relationship, or the way in which both parents and children influence each other’s development—including maintenance of difficult behaviors – via their behavioral responses (Singh et al., 2006). They aim to decrease parenting stress by developing more adaptive parenting skills, including reduced automatic reactivity and negative attentional bias (Bögels & Restifo, 2014; Friedmutter, 2015). This in turn may beneficially influence the bi-directional relationship between parenting stress and child challenging behavior (Neece et al., 2012). In a recent meta-analysis (Burgdorf et al., 2019), MBIs for parents conferred small to moderate (*g* = 0.34–0.53) immediate and maintained improvements on parenting stress. More recently, mindful parenting programs have also been developed, which differ from MBIs through application of established mindfulness interventions solely to a parenting context.

Mindful parenting refers to an ongoing process of mindfulness in relation to the parent’s subjective experience, their parenting interactions, and their child (Kabat-Zinn & Kabat-Zinn, 1997). The Mindful Parenting Program (MPP; Bögels and Restifo, 2014) is a mindfulness-based program for parents based on traditional MBI models (e.g., mindfulness-based stress reduction [MBSR]; Kabat-Zinn, 1990). Please note that the term MPP is used in relation to the program developed by Bögels and Restifo, whereas the term ‘mindful parenting program’ is used in reference to mindful parenting interventions more broadly. In the MPP, parents attend 3-hour group sessions over 8-weeks. The MPP includes conventional mindfulness (e.g., body scan) and self-compassion (e.g., self-compassion break, soothing rhythm breathing) practices, parenting education (e.g., rupture and repair), and home practice, which are all applied to the context of parenting. For example, the common practice of mindfully eating a raisin from MBIs teaches people to observe and notice as if for the first time (Kabat-Zinn, 1990); in the MPP, parents are invited to observe their child as if for the first time, without judgment and preconceptions (Bögels and Restifo, 2014), In addition, established practices like mindful self-compassion break (Germer & Neff, 2019) are utilized by parents when they are experiencing distress in the context of a difficult parenting interaction.

Emerging research provides evidence for the effectiveness of the MPP in clinical settings with respect to improved parent and child wellbeing, behavior, and social outcomes. A quasi-experimental study of parent participants (i.e., nonrandomized waitlist control; *N* = 86) referred to secondary child mental health care found improvements in parent and child internalizing symptoms, child externalizing symptoms, parental stress, parenting and co-parenting (Bögels et al., 2014). In an uncontrolled study of 70 parents of children diagnosed with a neurodevelopmental or mental health condition, parents experienced reduced psychopathology, increased mindful parenting and general mindful awareness, as well as improvements in their children’s internalizing, externalizing, and attention problem symptoms (Meppelink et al., 2016). The fact that parents’ mindful parenting, but not their general mindfulness, predicted child psychopathology suggests the value of this specific training focus (Emerson et al., 2019; Meppelink et al., 2016). A waitlist-controlled study reported the MPP was also effective for parents of children with Attention Deficit-Hyperactivity Disorder (ADHD), including reductions in parenting stress and problematic parenting interactions (Van der Oord et al., 2012).

There is scant research investigating the MPP’s application in preventative contexts; in an initial investigation into the efficacy of the MPP in nonclinical populations, improvements (small to medium effect size) for a number of parent (stress, overreactivity, mindful parenting, wellbeing and partner relationship) and child (behavior problems and wellbeing) outcomes were found (Potharst et al., 2018). This suggests that the beneficial effects of the MPP may be generalizable to community populations and may thus contribute to proactive approaches to the wellbeing of families (Potharst et al., 2018).

## Increasing Accessibility

The standard length and duration of an MBI – including the MPP – is 8 weeks of weekly 2- to 3-hour group sessions, with participants asked to complete approximately 45 minutes of daily home practice (Crane et al., 2017). Given the time and energy required from participants, feasibility of MBIs, particularly in a parenting context, may be problematic (Potharst et al., 2019). Reductions to program intensity (e.g., reduced home practice time) and provision of flexible formats (e.g., online, shorter duration programs) may improve program feasibility for parents. Brief interventions confer advantages including relative efficiency of cost and time; mitigation of geographical barriers to access; subsequent improved treatment adherence; and greater reach, compared to individual therapy (Spijkerman et al., 2016). In relation to parenting approaches, brief interventions may also function as a “foot in the door” for parents to access more individualized and/or extensive therapy services (Coakley & Barber, 2012).

Given that MPP research is emerging, and is based on established MBI protocols and research, it is important to consider MBI factors when reviewing the rationale for the current MPP study. For example, the optimal ‘dosage’ of mindfulness-based programs is not yet known. In a review of MBSR, Carmody and Baer (2009) noted no association between in-class hours and effect size of improved psychological outcomes for both clinical and nonclinical populations. Furthermore, even short-term experimental mindfulness inductions (range 5–25 minutes) can improve emotion regulation without the larger commitment of a full MBI program (Leyland et al., 2019). In a feasibility trial of a 6-week MBI for parents, with 1.5 hours per week sessions, (9 total contact hours, 10 minutes daily home practice), Lo et al., 2017 reported significant improvements (small to moderate effect sizes) in parenting stress and depression, especially for those experiencing severe symptoms. Given the need for preventative mental health approaches, and the limited time and financial resources available to many families, brief psychological interventions warrant investigation (Jorm et al., 2017).

In addition to providing shorter interventions, another adaptation to the MPP to improve accessibility may be to deliver the program in an online format. In a qualitative investigation of facilitators and barriers to engagement in a mindful parenting intervention, Ruuskanen, Leitch, Sciberras, and Evans (2019) reported that content accessibility, efficiency (i.e., brief program content and mindfulness practices), and support and ‘check-ins’ from peers and trainers were key perceived facilitators to program engagement by parents. In relation to parenting, an uncontrolled feasibility study investigating a 3-day online mindful parenting program for parents of preschoolers resulted in significant reductions in psychological distress and parenting stress (Van Gordon & Chapman, 2018). In a randomized controlled trial (RCT; *N* = 76; waitlist control group *n* = 33), Potharst et. al., 2019 assessed a self-directed, online version of the MPP, for mothers of children aged three to four years with elevated levels of parenting stress. The online MPP consisted of 8 weekly sessions, each of which included video content with formal meditations and mindfulness exercises, psychoeducation about mindful parenting, and home practice. The sessions were designed to take between 35 – 50 minutes to complete. The authors reported significant improvements in depression and anxiety (small to medium effect size) from participating mothers at post-treatment and follow-up (10 weeks post-intervention), and improvements in some aspects of parenting stress (small effect size) at follow-up. Thus, brief and online MPPs may provide a convenient and effective option.

## The Present Study: Feasibility of a Brief Online MPP

In the current pilot study, we aimed to assess the feasibility, including initial efficacy, of a brief-format MPP – the Two Hearts program – that comprises four online modules for a community population of parents. Consistent with published guidelines on determining the feasibility of novel interventions (Bowen et al., 2009; Thabane et al., 2010), the primary objectives were to determine the feasibility of the program, acceptability to parents, and initial efficacy on outcomes of parenting stress and general distress. Research questions were:

1. How feasible and acceptable is the program?

2. Does the program reduce parenting stress and parent general distress?

As a novel approach, we were interested in what level of participation was feasible for parents, to inform future design/refinement of the program. We specifically assessed rate of retention, participation in activities, including online content (e.g., online video engagement) and home practices. While a guideline of 20 minutes home practice per day 5 days per week was encouraged, an exploratory approach for evaluating program adherence was utilized, given home practice variability reported in previous online MPP research (Potharst et al., 2018). Parent evaluation of the program was used to evaluate acceptability with an expectation that 80% of parents would endorse the program as acceptable (as per Bögels & Restifo, 2014). Initial efficacy was assessed on the primary outcome measures of parenting stress and parent general distress. Based on previous research, we predicted that participants would report significant improvements in parenting stress and general distress following completion of the Two Hearts Program. Effects on secondary outcomes, namely mindful parenting, general mindful awareness, and self-compassion, were also exploratory and no specific hypotheses made a priori.

## Materials and Method

### Design and Context

A single case design was used, with data collected at three time points; T1 (one week prior to intervention), T2 (immediately post-intervention), and T3 (four weeks after completion of the intervention). Dependent variables were program feasibility and acceptability, parenting stress, parent general distress, mindful parenting, general mindful awareness, and self-compassion. Ethical approval was obtained from the Griffith University Human Research Ethics Committee (Approval Number 2019/853) and all participants gave signed informed consent. The study was conducted April to June of 2020 in Australia; during initial Covid-19 restrictions (e.g., mandatory home-based isolation, school closures). An unplanned semi-structured telephone interview was consequently added by the first author in response to these unexpected events, with program participants to determine their preferences for program format (e.g., live or pre-recorded sessions, video duration, program duration) moving forward within the bounds of the unexpected restrictions. This led to moving the initially planned in-person program to an online asynchronous program which recruited families deemed acceptable in telephone interviews with variations approved by the ethical approval board prior to implementation. This telephone interview was undertaken to improve program acceptance and feasibility of the revised format in response to restrictions arising from the pandemic.

### Participants

Participants (*N* = 6) were recruited from broad community advertisements targeting parents of preschoolers with anxiety for inclusion in another MPP study. Parents who did not meet criteria for that study (i.e., children were older than preschool age, children did not have clinical anxiety) were offered the opportunity to participate in the current study. Inclusion criteria for the current study were being an adult parent aged 18 or older of children aged 5–16 years old, with proficient English, who were able to access online materials and commit to the program practices, and who were not participating in another parenting training program. Pseudonyms have been used to protect participant confidentiality. See Table 1 for a description of participants. Parents were aged between 32–46 years, with children aged between 4–9 years. All parents were educated to tertiary level, four to post-graduate degree level. All parents were married with two children. Most participants did not practice meditation prior to the study.

**Table 1:** Description of Participants

	*Age*	*Gender*	*Ethnicity*	*Education*	*Employment*	*Meditation (minutes)*	*Parent Dx*	*Relationship Status*	*Number of Children*	*Relationship to Child*	*Child’s Age*	*Child Dx*	*Annual Household Income* *($ 000* *s)*
Frank	42	Male	Caucasian	PG Degree	Self-Employed	0	N/A	Married	2	Biological Parent	9	N/A	90–180
Sally	32	Female	Caucasian	PG Degree	FT Paid Employment	120	PND	Married	2	Biological Parent	7	N/A	90–180
Elizabeth	44	Female	Asian	PG Degree	FT Student	0	N/A	Married	2	Biological Parent	8	N/A	90–180
Rebecca	46	Female	Caucasian	Bach. Degree	FT Student	0	N/A	Married	2	Biological Parent	9	ADHD	90–180
Linda	39	Female	Caucasian	Bach. Degree	Self-Employed	30	“CPTSD”	Married	2	Biological Parent	5	“ADD”(I)	90–180
Vanessa	34	Female	Asian	PG Degree	PT Paid Employment	0	N/A	Married	2	Biological Parent	4	N/A	Prefer not to answer

*N/A* not applicable, *PG* Post-graduate. *Bach* Bachelor’s. *FT* full-time. *PT* part-time. *Meditation* minutes of meditation practice engaged in each week. *Parent Dx* parent’s self-report psychiatric diagnosis/es in their own words. *PND* Post-Natal Depression. *CPTS* not specified but interpreted to refer to Complex Post-Traumatic Stress Disorder. *Child’s Age* the age of the child who the parent is focusing on during the program (i.e., the one with whom they have the most challenging interactions). *Child Dx* parent’s report of child psychiatric and/or developmental diagnosis/es. *ADD(I)* parent’s report of “ADD Inattentive”, interpreted to refer to Attention Deficit Hyperactivity Disorder (Inattentive type)

### Measures

#### Program Feasibility and Acceptability

An adapted version of the MPP evaluation form (Bögels & Restifo, 2014), was used to assess participants’ satisfaction and engagement with the current program.

#### Parenting Stress

The Parenting Stress Index Short Form Fourth Edition (PSI–SF; Abidin, 2012) was used to measure parenting stress. A total stress score is yielded from three domains that measure parental distress (PD), parent-child dysfunctional interaction (PCDI), and difficult child (DC). The PSI–SF demonstrates good validity and reliability (PD α = 0.90, PCDI α = 0.89, DC α = 0.88, total score α = 0.82; Abidin, 2012).

#### Parent General Distress

The Hospital Depression and Anxiety Scale (HADS; Zigmond & Snaith, 1983) is a self-report measure of anxiety and depression. The HADS demonstrates adequate validity and internal consistency (mean anxiety subscale α = 0.83, mean depression subscale α = 0.82; Bjelland et al., 2002; Mykletun et al., 2001).

#### Mindful Parenting

The Interpersonal Mindfulness in Parenting Scale (IM-P; de Bruin et al., 2014) was used to assess mindful parenting. The 31-item English version of the IM-P was administered to participants (Duncan et al., 2009), however, as this scale has not yet been validated, scoring from the 29-item IM-P (de Bruin et al., 2014) was used to calculate the total score used for this study. The IM-P is a reliable and valid measure of mindful parenting (α = 0.85; de Bruin et al., 2014).

#### General Mindful Awareness

The 15-item version of the Five Facet Mindfulness Questionnaire (FFMQ-15; Baer et al., 2006; Baer et al., 2012) was used to assess general mindful awareness. There are five subscales – observing, describing, acting with awareness, nonjudging of inner experience, and nonreactivity to inner experience – and a total score. The FFMQ-15 has demonstrated adequate validity and internal consistency (subscales α = 0.64 - 0.80; Gu et al., 2016). As recent research has indicated that the observing subscale is problematic and should be excluded from analyses of intervention effects (Gu et al., 2016; Sherwood et al., 2020), observing items were excluded from the total.

#### Self-compassion

The Self-Compassion Scale-Short Form (SCS – SF; Raes et al., 2011) is a 12-item measure of self-compassion. The total score was used, as recommended for improved reliability (α = 0.86; Raes et al., 2011).

## Procedure

### Data Collection

Data was collected at three time points (i.e., T1, T2, T3 as noted above). Program evaluation was administered at T2 and T3. All assessments were completed via online surveys (Lime Survey; participants were sent links to the surveys via email).

### Intervention

Two Hearts program content was selected from the MPP (Bögels & Restifo, 2014) based on considerations of: fundamental mindfulness (Crane et al., 2017) and self-compassion training; elements deemed most salient for the reduction of parenting stress; previous participants’ ratings of the value of various MPP exercises (Bögels et al., 2014; Bögels & Restifo, 2014); and which elements were best suited to a limited contact, online training program (e.g., longer/more complicated practices were excluded). The authors consulted with MPP program developer (Susan Bögels) on proposed content which was used with permission. Participant telephone interview data was used to inform the final format and design of the program.

The outline of the Two Hearts program is presented in Table 2. Modules were sent to parents on a weekly basis via email for 4 weeks. The introductory module consisted of a PDF document describing mindfulness and self-compassion, and their relevance to parenting stress, with an accompanying audio file of Body Scan meditation. All subsequent modules consisted of a series of short videos hosted on a dedicated YouTube channel, readings from the Two Hearts Parent Workbook, audio files of mindfulness practices, and directed home practices. The Two Hearts Parent workbook was a PDF document that included key workshop content based on the MPP handouts and exercises (Bögels & Restifo, 2014), with some exercises adapted for the Two Hearts program (e.g., Self-Compassion Break, adapted from Germer & Neff, 2019). Two optional individual support phone or Zoom sessions (30-minute duration) were offered to parents after Modules 1 and 3. Parents were also sent text message reminders (e.g., *Good morning, how’s your mindfulness practice going?*) twice per week during the intervention period and once per week in the follow-up period. Program videos were delivered by a provisional Psychologist, with training in mindfulness- and compassion-based psychotherapeutic approaches, 10 years established mindfulness practice and meditation, 7 years’ experience as an Iyengar Yoga student, and completion of a MPP workshop.

**Table 2:** Two Hearts Program Outline

Module Format	Module Title	Themes	Mindfulness Practice	Specific Mindful Parenting Exercise	Home Practice
**Introductory Module** PDF document, audio file, home practices (approx. 1.75 hours)	Introductory Module	Basic principles of mindfulness and compassion; Relevance of mindfulness and compassion in parenting stress; Introduction to home practices	Body Scan (Williams, Teasdale, Segal, & Kabat-Zinn, 2007) Informal mindfulness practice (i.e., mindful coffee)	N/A	15 min guided Body Scan meditation and informal mindfulness (1 pd, 5 times pw)
**Module 1** Series of four short videos, audio file, program workbook, home practices (approx. 3 hours)	Mindful Awareness in Parenting	Introduction to Mindful Parenting and orientation to program; The evolution of parenting stress; Body awareness and the stress response; Finding a Breathing Space	3-min Breathing Space	Imagination: parenting stress exercise	Parent Workbook readings; 15 min guided Body Scan practice (1 pd, 5 times pw); Breathing Space (2 pd, 5 times pw); use of optional mindfulness home practice record
**Module 2** Series of four short videos, home practices (approx. 3 hours)	Mindful Parenting in Action	Automatic pilot parenting; Body awareness and Mindful Movement; Rupture and Repair	Mindful Movement	Imagination: Rupture and Repair exercise	Parent Workbook readings; 15 min guided Body Scan practice (1 pd, 5 times pw); Breathing Space (2 pd, 5 times pw, and use during stressful interaction); Parenting Stress Diary (1-3 entries pw); Rupture and Repair process at home; use of optional mindfulness home practice record
**Module 3** Series of two short videos, audio file, *Useful Resources* PDF, home practices (approx. 2 hours)	Compassion in Parenting	Compassion, Stress and the Body; Growing Self-Compassion	Soothing Rhythm Breathing (Gilbert, 2009); Self-Compassion Break (Neff & Germer, 2013)	Imagination: compassion for self during parenting stress exercise	Parent Workbook readings; ‘*What Do I need?*’ reflective exercise (Bögels & Restifo, 2014); Individualized commitment practice based on practices learned to date; inclusion of Module 3 Self-Compassion practices; use of optional mindfulness home practice record
**Optional Individual Support Session** Zoom videoconferencing or phone (up to 30 minutes, twice during the program during week of Module 1 and Module 3)	N/A	Review of individual mindfulness practice and mindful parenting scenarios; Positive reinforcement of parent efforts; Collaborative problem-solving of barriers and challenges in mindfulness practices	N/A	N/A	N/A

*N/A* not applicable, *pw* per week, *pd* per day

### Data Analyses

Descriptive analysis of participant retention, online video engagement (i.e., total views, unique views and percentage watched), and home practice data were used to evaluate what was feasible. To evaluate acceptability, Item 1 of the Program Evaluation (“*Did you feel you got something of lasting value or importance as a result of taking the training?”)* was analyzed using descriptive statistics (threshold set that 80% of parents would endorse as per Bögels & Restifo, 2014).

To evaluate effectiveness, individual level analyses were conducted on the preliminary outcome measures using the reliable change index (Jacobson & Truax, 1992) to evaluate if changes were statistically reliable at an individual level. Clinically significant change was defined in the current study as moving from one clinical range to another (e.g., clinical cut-off to elevated or normal range), according to normative data. Only descriptive statistics for secondary outcomes are reported due to the small sample size.

## Results

### Feasibility and Acceptability

All parents remained engaged in the study to completion at T3 (i.e., 100% retention). Engagement with the online videos is summarized in Table 3. The number of unique views indicates the proportion of the sample of participants that engaged with each video. The duration watched indicates the percentage of the total video time that viewers watched on average. The highest number of unique views (5) was recorded for the mindful movement video (module 2), which had the lowest duration watched (76.3%). The lowest number of unique views (2) was recorded for the two compassion videos that comprised module 3, which had a high duration watched (100%, 99.8%). The second lowest duration watched was recorded for the rupture and repair video (80%). Engagement was relatively consistent across modules 1 and 2, with at least 67% of participants watching each video, and most videos watched in full. All video views occurred within the respective week for that module between T1 and T2; no views were recorded between T2 to T3. Videos were generally rated favorably (T2 *M* = 8.33, range 5–10, where *not important at all* = 1 and *extremely important* = 10). Module 3 videos were the least watched; the program content to which these videos related (i.e., compassion) was one of the most highly rated (T2 *M* = 9.67).

**Table 3:** Participant Engagement with Video Materials

	Total views	Unique views (% of sample)	Duration watched (%)
**Module 1**			
*Video 1. Introduction*	4	4 (67%)	100%
*Video 2. Evolution of parenting stress*	4	4 (67%)	106%
*Video 3. Your parenting stress*	4	4 (67%)	101%
*Video 4. Finding a breathing space*	4	4 (67%)	97%
**Module 2**			
*Video 1. Autopilot*	4	4 (67%)	87.4%
*Video 2. Mindful movement*	6	5 (83%)	76.3%
*Video 3. About mindful movement*	5	4 (67%)	112%
*Video 4. Rupture and repair*	4	4 (67%)	80%
**Module 3**			
*Video 1. Compassion, stress and the body*	2	2 (33%)	100%
*Video 2. Growing self-compassion*	2	2 (33%)	99.8%

>100% duration watched indicates that participants rewound and rewatched sections of video. ‘Total ‘views is the total number of video views, whereas ‘unique views’ is the number of individual participants who viewed the video

Program adherence varied considerably. Parents were encouraged to complete 20 minutes of home practice per day, 5 days per week. Two parents practiced 10 to 15 minutes per week on average (Elizabeth and Vanessa) and the remaining parents practiced 40 to 50 minutes per week. At T3 adherence had declined, however, half of the sample continued to practice 30 to 50 minutes per week in the follow-up period.

Program evaluation results are presented in Table 4. Program acceptability was high; at T2 and T3 there was 100% endorsement of item *Do you feel you got something of lasting value or importance as a result of taking this training?*, and a high overall training importance rating, although this decreased somewhat at T3. In addition, all parents reported that they have become more conscious as a result of the training (Item 3) and have an intention to continue with mindful parenting practice (Items 2, 4, 5). These results were sustained at follow-up, however, one parent (Elizabeth) reported that she had not made any changes as a result of the training at T3 (Item 2). In general, most parents rated all components as important (≥7); Frank and Elizabeth assigned lower ratings than other parents across many program elements, and this was also reflected in their overall rating of training importance (average across T2 and T3 Frank = 6.5, Elizabeth = 7.5). While Frank, Sally, and Vanessa gave lower ratings to Text Reminders (3–6), other parents rated this element highly (10).

**Table 4:** Feasibility and Acceptability – Program Evaluation

General Program Evaluation	T2			T3		
	*Yes*		*No*	Yes		*No*
1. Do you feel you got something of lasting value/importance from the program?	6		0	6		0
2. Do you intend to make (T3 Have you made) any changes (e.g., lifestyle, parenting) as result of training?	6		0	5		1
3. Did you become more ‘conscious’ as a result of the training?	6		0	6		0
4. Is it your intention to keep practicing the mindfulness exercises (e.g., Body Scan, 3-min Breathing Space)?	6		0	6		0
5. Is it your intention to keep practicing to be conscious in daily parenting life?	6		0	6		0
	*Mean*	*SD*	Range	Mean	*SD*	Range
6. Has the training been sufficient to move on with your life as a parent?	0.67	0.52	0-1	0.67	0.52	0-1
7. How many times do you pay attention to your child in moments when you are together, compared to before the training?	3.00	0.63	2-4	2.67	1.03	1-4
8. How many times per week, on average, did you do the mindfulness practices during the 3-week period between the Introductory Module and Module 3?	2.50	0.55	2-3	-	-	-
9. How many minutes per week, on average, did you do the mindfulness practices during the 3-week period between the Introductory Module and Module 3?	32.50	16.00	10-50	-	-	-
10. How many times per week, on average, did you do the mindfulness practices during the previous 3 weeks since Module 3?	-	-	-	1.67	1.03	0-3
11. How many minutes per week, on average, did you do the mindfulness practices during the previous 3 weeks since Module 3?	-	-	-	22.83	21.91	0-50
**Importance Ratings of Program Components**						
12. Training Importance	8.33	1.00	7-10	7.83	1.33	6-10
13 Introductory Module	7.50	2.26	5-10	8.50	1.38	7-10
14. Program Pacing	8.67	1.21	7-10	7.67	2.58	4-10
15. Online Format	8.83	1.84	6-10	8.33	2.25	5-10
16. Informal Mindfulness	7.67	2.25	4-10	8.17	1.94	5-10
17. Body Scan	7.83	2.23	4-10	7.50	2.95	2-10
18. Mindful Movement	6.67	1.21	5-8	6.00	2.68	1-8
19. 3-Minute Breathing Space	7.83	2.86	3-10	8.17	2.14	5-10
20. Self-Compassion Break	-	-	-	7.33	1.75	5-10
21. Soothing Rhythm Breathing	-	-	-	8.33	1.97	5-10
22. Awareness in Daily Parenting	9.33	1.03	8-10	8.17	1.94	5-10
23. Rupture and Repair	9.50	0.84	8-10	7.83	1.94	5-10
24. Compassion for Self and Child	9.67	0.82	8-10	8.33	2.25	5-10
25. Program Videos	8.50	2.07	5-10	8.00	2.37	5-10
26. Audio Files	9.67	0.82	8-10	8.17	2.23	5-10
27. Parent Workbook	8.17	2.56	4-10	7.83	1.94	5-10
28. Optional Zoom Sessions	5.50	3.27	1-10	7.67	2.34	4-10
29. Supportive texts	8.17	2.04	6-10	7.33	3.20	3-10

Items 1 to 6 answer option: *no* = 0, *yes* = 1. Item 7 answer option: *less than before* = 1, *as much as before* = 2, *more than before* = 3, *much more than before* = 4. Item 8 answer options: *never* = 0, *1-2 times* = 1, *3-4 times* = 2, *5-7 times* = 3. Items 12 to 29 response options: *not important at all* = 1 to *extremely important* = 10

Four of six parents (individual participants are described below) utilized the optional support sessions. These sessions, including their individual ‘opt in’ nature, were included in the program in response to parents’ requests, and as part of the adjustments made due to COVID-19. This program component was thus designed to improve program acceptability. In terms of uptake, one session occurred in the week of Module 1 (Vanessa). Three sessions occurred in the week of Module 3 (Vanessa, Frank, and Elizabeth). A 30-minute unscheduled phone call with Linda also took place in the week following Module 3. Themes of these sessions included discussion of: program adherence (e.g., which practices had been completed, practice preferences, mismatch between program aim and scope and parent expectations (Frank)); barriers to practice (e.g., remembering or finding time to do practices, falling asleep during Body Scan or finding it too long); adjusting unhelpful expectations of self in regards to mindfulness skills and implementation; tailoring practices to specific parenting challenges; clarification of mindfulness aims (e.g., differentiating mindfulness and relaxation) and processes (e.g., importance of nonjudgment and nonreactivity).

### Initial Effects

Table 5 shows scores on the primary outcome measures of parenting stress and general distress, and secondary outcome measure of mindful parenting, for each parent at each timepoint, as well as reliable change index (RCI).

**Table 5:** Parenting Stress, Parent General Distress and Mindful Parenting scores and Reliable Change Index

	Frank					Sally					Elizabeth				
	T1	T2	T3	RCI _1.2_	RCI _1.3_	T1	T2	T3	RCI _1.2_	RCI _1.3_	T1	T2	T3	RCI _1.2_	RCI _1.3_
**Parenting Stress**															
PD	42/66	32/55	29/52	−2.46*	−3.13*	46/70	35/58	23/46	−2.68*	−5.37*	35/58	39/63	40/64	1.12	1.34
PCDI	30/57	26/53	26/53	−0.85	−0.85	41/70	25/51	24/50	−4.04*	−4.26*	40/69	44/74	42/71	1.06	0.43
DC	46/71	38/62	41/65	−1.84	−1.23	43/68	38/62	28/50	−1.23	−3.68*	41/65	47/72	50/76	1.43	2.25
*Total*	118/66	96/57	96/57	−2.84*	−2.84*	130/71	98/58	75/48	−4.10*	−7.26*	116/65	130/71	132/72	1.89	2.21
**Parent General Distress**															
Anxiety	8	3	6	−2.21*	−0.88	13	6	4	−3.10*	−3.98*	3	10	19	3.10	7.08
Depression	8	7	6	−0.48	−0.96	9	5	2	−1.92	−3.37*	5	8	15	1.44	4.81
*Total*	16	10	12	−1.89	−1.26	22	11	6	−3.47*	−5.05*	8	18	34	3.15	8.20
**Mindful Parenting**	82	98	100	3.07*	3.45*	87	90	104	0.57	3.26*	65	78	55	2.49*	−1.92
	**Rebecca**					**Linda**					**Vanessa**				
	T1	T2	T3	RCI _1.2_	RCI _1.3_	T1	T2	T3	RCI _1.2_	RCI _1.3_	T1	T2	T3	RCI _1.2_	RCI _1.3_
**Parenting Stress**															
PD	29/52	16/38	14/36	−3.13*	−3.58*	37/61	39/63	41/65	0.45	0.89	16/38	18/40	19/41	0.45	0.67
PCDI	34/62	17/42	21/47	−4.26*	−3.19*	30/57	25/51	28/55	−1.28	−0.43	29/56	26/53	21/47	−0.64	−1.91
DC	42/66	26/48	28/50	−3.68*	−3.27*	44/69	42/66	41/65	−0.61	−0.82	26/48	34/57	24/46	1.84	−0.41
*Total*	105/61	59/42	63/44	−5.99*	−5.36*	111/63	106/61	110/63	−0.63	0	71/47	78/50	64/44	0.95	−0.95
**Parent General Distress**															
Anxiety	10	3	3	−3.10*	−3.10*	10	18	17	3.54	3.10	9	8	8	−0.44	−0.44
Depression	2	0	0	−0.96	−0.96	3	9	7	2.88	1.92	1	3	0	0.96	−0.48
*Total*	12	3	3	−2.84*	−2.84*	13	27	24	4.42	3.47	10	11	8	0.32	−0.63
**Mindful Parenting**	93	115	116	4.21*	4.41*	107	106	106	−0.19	−0.19	88	79	90	−1.72	0.38

*Parenting Stress* Parenting Stress Index – Short Form (PSI – SF). *PD* Parental Distress subscale score. *PCDI* Parent-Child Dysfunctional Interaction subscale score. *DC* Difficult Child subscale score. PSI – SF scores are presented as *raw score/T-score*. T-scores used for calculation of RCI (i.e., *M* = 50, *SD* = 10; PD α = 0.90, PCDI α = 0.89, DC α = 0.88, Total Score α = 0.82; Abidin, 2012). Parent General Distress: Hospital Anxiety and Depression Scale (HADS). HADS normative data (Anxiety subscale: *M* = 6.14, *SD* = 3.76, α = 0.82; Depression subscale: *M* = 3.68, *SD* = 3.07, α = 0.77; Total score *M* = 9.82, *SD* = 5.98, α = 0.86) from Crawford, Henry, Crombie, and Taylor (2001). HADS subscale cut-offs: 0–7 = normal, 8–10 = mild, 11–21 = clinical (Zigmond & Snaith, 1983). Mindful Parenting: Dutch version of the Interpersonal Mindfulness in Parenting Scale total score. Normal *M* = 109.29, *SD* = 11.13, α = 0.89 (de Bruin et al., 2014). RCI = Reliable Change Index (e.g., RCI _1.2_ = RCI for T1 to T2). HADS and IM-P normative data was used to calculate RCI. RCI is significant (i.e., reliable change has occurred) if value is >1.96 (Jacobson & Truax, 1992). Significant RCI values are indicated by*

#### Parenting Stress

At T2 and T3, three of six parents (Frank, Sally, Rebecca) reported reliable improvements in parenting stress, and two of these (Frank and Sally) also achieved clinically significant change, moving from the clinical to normal range. As Rebecca’s scores were in the normal range across all three time points, clinical change was not possible. Linda and Vanessa did not demonstrate reliable beneficial effects, and both were in the normal range across time points. Elizabeth experienced reliable worsening of parenting stress at T3, and her T1 clinical range parenting stress score was maintained at follow-up. Both Elizabeth and Linda reported (i.e., Program Evaluation Item 11 and Zoom/phone reports) that they had experienced significant personal stressors in the evaluation period.

#### Parent General Distress

At the total score and anxiety subscale score levels, two parents (Sally and Rebecca) of six achieved reliable reductions in general distress at T2 and T3 (see Table 4). Sally – the only parent who had a general distress score in the clinical range (anxiety only) at T1—also demonstrated a clinically significant change (reduced to normal range) at T2 and T3. No other parents achieved clinical change and they were in the normal range for all scores. Depression scores were minimally impacted during the program; Sally alone had a reduced depression score at T3. Some parents’ general distress increased during the program. At T3 Elizabeth’s anxiety and depression scores had reliably worsened and deteriorated from the normal to clinical range. At T2 and T3, Linda’s anxiety score demonstrated reliable worsening, and moved from the mild to the clinical range. As noted previously, Elizabeth and Linda had reported significant personal stressors in the evaluation period which may explain these changes. In general, however, most parents’ anxiety and depression scores changed in the expected direction indicating improvement.

#### Mindful Parenting

At T2, three parents (Frank, Elizabeth, Rebecca) demonstrated reliable beneficial change in their mindful parenting. At T3, half the sample continued to show positive change in their mindful parenting, however, one parent (Elizabeth) who had initially shown improvements at T2 no longer did so at T3, and one other parent (Sally) who did not show reliable change at T2, did at T3.

#### General Mindful Awareness

Three parents (Frank, Rebecca, and Linda) showed an increase in general mindful awareness, and three showed a decrease (Sally, Elizabeth, and Linda), from T1 to T2. At T3, five parents had experienced an increase in general mindful awareness with only Elizabeth not showing a reduction compared to T1.

#### Self-compassion

Four parents (Frank, Sally, Rebecca, and Vanessa) experienced a beneficial increase in self-compassion, and two (Elizabeth and Linda) experienced a decrease, from T1 to T2. At T3, all parents except Linda demonstrated an increase in self-compassion, who experienced a decrease, compared to T1.

## Discussion

The current study delivered a brief online MPP training, the Two Hearts program, over a 4-week period to a community sample of parents. The study aimed to assess program feasibility and acceptability to parents, as well as initial effects on parenting stress and distress, and effects on the secondary outcomes of mindful parenting, general mindful awareness, and self-compassion. The results indicated that program acceptability was adequate. Further, levels of feasible engagement in the program are outlined, and preliminary findings indicate the program may deliver beneficial reductions in parenting stress and distress.

### Feasibility and Acceptability

A key outcome was that parents found the program acceptable. At T3, five of six parents (all except Frank) reported they had found lasting value in the program and had made subsequent changes to their parenting/lifestyle. With nil drop out from the study, retention was also good, and compares favorably with Potharst et al., 2019 online MPP, which achieved 86% retention at T3 for the intervention group. The high retention of the current study thus highlights the potential for brief, self-paced online program approaches. Adherence was variable, however, video engagement was not 100%, and so indicates that only a proportion of participants watched the video content of the program. Video engagement was generally consistent across modules 1 and 2, with stability in number of unique views and duration watched. Engagement dropped substantially for module 3, with only two parents watching each video. It is not possible to comment on whether video engagement across the program was stable for individual participants (i.e., to track individual engagement across the program). The aggregate data suggests that the video content was not utilized as a core part of the program for all participants, as was intended. No previous studies have reported engagement data for online self-guided components of mindful parenting programs, and so comparison to broader literature is not possible. In their online MPP study, Potharst et al. (2019) reported that participating mothers completed an average of 3.8 from the available 8 online sessions (47%). The authors however did not provide further data relating to engagement with the online sessions and variance across the program.

In relation to home practice, results varied considerably across participants and time points. For example, at T2, four parents practiced for a duration of 40–50 minutes (Sally, 50 minutes, Frank, Rebecca, and Linda, 40 minutes), however, at T3, home practice had generally reduced; while two parents completed at least 45 minutes practice per week (Frank and Linda), two parents reported negligible practice (0–2 minutes). Although parents’ home practice was less than the guideline suggested to them, it was higher than Potharst et al., 2019 online program (*N* = 43, average 14.94 minutes per week, range 0–120 minutes). In general, given the results of the current study, a feasible amount of home practice for an online MPP appears to be significantly less than traditional MBIs (Crane et al., 2017). Program acceptability was good and comparable to the traditional MPP format (Bögels & Restifo, 2014; Potharst et al., 2018). Program retention and acceptability suggests that parents were motivated to complete the Two Hearts program.

### Initial Effects

Partial support for predicted initial effects were found. Reductions in parenting stress at T2 and T3 were achieved by half the sample (Frank, Sally, Rebecca). Reductions in parent general distress at T2 and T3 were also achieved in three of six parents’ anxiety scores (Frank, Sally, Rebecca), however, only two parents (Sally and Rebecca) demonstrated reliable anxiety reductions at T3. Further, only two parents experienced significant reductions in total distress scores at T2 and T3 (Sally and Rebecca). Previous research has indicated that there is a direct link between amount of home practice and whether intervention effects are achieved: the more practice an individual does, the better their outcome (e.g., Burgdorf et al., 2019; Carmody & Baer, 2008). In the current study, parents who completed more practice tended to improve; two of three parents (Elizabeth and Vanessa) who did not experience beneficial effects from the training also reported the lowest practice. Thus, it is possible that adherence in the current study may have impacted program effects for parents. These findings suggest future programs could aim for at least 40 minutes home practice per week, based on amounts by parents (Frank and Sally) showing gains, and research into facilitators and barriers of home practice to inform future program development would be valuable to support outcomes in future research.

Mixed findings may also be due to only one parent having a baseline general distress score in the clinical range, and only for the anxiety subscale. Compared to other MPP studies that have investigated clinical samples and found medium effect sizes for parenting stress and psychopathology (Bögels et al., 2014; Meppelink et al., 2016), there could be less room for movement from more adaptive scores at baseline.

A final explanation for the mixed results is that both Linda and Elizabeth reported multiple acute stressors during the evaluation period. According to Elizabeth, one of the ways these stressors impacted her was in skills practice and development. While tailored support was provided in a video conferenced session at the end of the program, it is possible that she (and Linda) would have benefited from earlier and more targeted support. Previous research indeed, has found that targeted interpersonal support such as ‘check ins’ from peers and trainers were identified by parents as being useful for their program engagement (Ruuskanen et al., 2019). The necessity for some kind of ‘live’ support is not, however, a given. Potharst et al., 2019 online MPP was entirely self-paced, had no trainer or peer contact for participants, and yet reported significant beneficial outcomes for parents and their children. Further research is needed to determine what constitutes minimal engagement with online MPPs for parents to experience benefits (e.g. reduced stress, increased wellbeing), and indeed whether support from a trainer or therapist is required for some parents. Engagement is a known challenge in parenting program research, with attrition rates of 51% across studies (Chacko et al., 2016). The bias toward effectiveness, and limited reporting of vital feasibility data, limits the successful implementation of such approaches more widely.

Half the sample indicated improvements in mindful parenting at T3, however, results were variable. For example, Elizabeth, but not Sally, had increased mindful parenting scores at T2. In contrast, the scores on self-compassion and general mindful awareness were higher at T3 than T1 for five of the six parents with Linda and Elizabeth the exceptions at T3 for self-compassion and general mindful awareness respectively. While the lack of established norms combined with the small sample size precluded analysis of these secondary outcome scores (either as a group score or using the reliable change index) to determine statistical significance, this result provides initial evidence that improvements in self-compassion or general mindful awareness may also contribute to initial effects.

Initial effects for the Two Hearts program compare favorably to 8-week MPPs of more traditional and online formats. Preventative MPPs following a more traditional in-person delivery over 8-weeks have also demonstrated small reductions in parenting stress (Potharst et al., 2018). Potharst et al., 2019 reported that mothers of toddlers with elevated stress levels, who completed an 8-week online MPP showed improvements in parenting stress only at follow-up. In contrast to the current study, anxiety and depression reduced at post-intervention and follow-up, with medium effect sizes (Potharst et al., 2019).

### Limitations

The study was conducted in the midst of the COVID-19 pandemic, when a number of significant changes to people’s lives (e.g., mandatory isolation, home schooling) and related stressors (e.g., health anxiety, economic pressures) were present. It is unclear what impact this situation had on parents’ motivation and capacity to engage in the program, or the effects achieved. Sensitivity to change was a limitation of the measurements utilized in this study, for the community sample (i.e., the HADS is typically used in clinical populations). There is also a paucity of outcome measures in the field of mindfulness, and noted psychometric limitations from previous research (e.g., no psychometric evaluation of FFMQ for single case designs, poorly matched sample for the IM-P normative data, significant positive skew in HADS normative data) as well as an absence of normative data for secondary measures which precluded calculation of reliable change indexes for these measures.

The video engagement data is only aggregate and anonymized, and therefore does not provide an indication of individual participant engagement. Finally, it is unclear if program format, duration, or feasibility (or a combination of these elements), impacted some parent’s outcomes. For example, program feasibility may not have been sufficient to achieve adequate dosage (e.g., two of three parents who did not improve also did markedly less home practice). The small sample size also obscures an understanding of home practice feasibility. No parent was able to complete the home practice guideline (i.e., 20 minutes per day, five days per week) and, while some parents were able to complete around 45 minutes practice per week, others did not. While previous research has shown no association between MBI contact hours and effect sizes (e.g., Carmody & Baer, 2009), there is an indication that amount of practice is associated with beneficial change (Burgdorf et al., 2019; Carmody & Baer, 2008). Therefore, there is still likely to be a minimum dosage in relation to practice engagement, and variable adherence in the Two Hearts program may have meant that this was not achieved. Despite these limitations, the current pilot study provides useful information regarding feasibility of this online MPP, which can inform future large scale feasibility and efficacy trials.

### Implications and Future Directions

While the guidelines for home practice may not have been feasible in the current intervention design, nor the global pandemic context (which stretched many people’s practical and mental resources), it is notable that parents who *did* practice tended to report beneficial effects. Future research and implementation should thus consider supporting improved adherence. Given that most parents opted for the optional support sessions, more structured and facilitated support may be required to improve adherence, and thus feasibility. Inclusion of support sessions *early* in the program may help parents to: tailor their home practice to suit their needs, especially in the event of acute stressors; problem-solve barriers to practice; and promote accountability. Future studies could also support greater engagement through adding parental goals, more targeted practice plans, logs of practice, and group sessions or connection tools, such as online support/peer groups. Such strategies could enable facilitated mindful inquiry; this key mindfulness learning process was not sufficiently incorporated in the current program, and this may have impacted parents’ acquisition of mindfulness skills and subsequent outcomes (Crane et al., 2017). Further, these elements may facilitate *connection with peers* – one of the change processes of MBIs in general identified by Ridderinkhof, deBruin, Blom, Singh, and Bögels (2019; an MBI for children on the autism spectrum and their parents) and which may be particularly important for MPPs.

A novel – and important, given its online format – aspect of this study was the reporting of engagement with video material. The availability of such data provides an overview of how the sample of parents interact with this part of the program. Future research could track individual participant engagement information (e.g., ask parents to ‘subscribe’ to video content to enable recording of individual engagement analytics). Dose effects should also be explored in future research of brief, online MBIs for parents, which may support parents’ well-being, parenting skills, and other important outcomes, via an efficient, affordable, and accessible therapeutic option. Whilst initial efficacy and effects are important to determine, further feasibility research is also vital to contribute to the improved design and relevance of MPPs, and thus increase engagement and reach of such supports.

In terms of assessment of effects and particular populations, measures designed for nonclinical populations (e.g., Depression, Anxiety and Stress Scale, Lovibond & Lovibond, 1995) should be considered for future research with community samples. In addition, broader measures of outcome, such as quality of life, changes to parenting styles, or impacts on children’s wellbeing or child’s behavior in response to changes in parenting styles, could capture other potential benefits of the program (e.g., as per Potharst et al., 2018, 2019; Bögels et al., 2014).

## Conclusion

The Two Hearts program may be a feasible and acceptable option for parents and may deliver beneficial reductions in parenting stress and general distress for some parents. It remains unclear what role factors such as program adherence, dosage, sample and selection of outcome measures, and program format may have on intervention effects in a brief online MPP. There is also a need for sensitive measurement of broader well-being outcomes, to determine the potential benefits of a program like Two Hearts, and which populations may best be served by such an intervention. Future research in this area could support parents’ well-being, parenting skills, and other important outcomes, via an efficient, affordable, and accessible therapeutic option.
